# DNA promoter methylation as a diagnostic and therapeutic biomarker in gallbladder cancer

**DOI:** 10.1186/1868-7083-4-11

**Published:** 2012-07-13

**Authors:** Pablo Letelier, Priscilla Brebi, Oscar Tapia, Juan Carlos Roa

**Affiliations:** 1 Universidad de La Frontera, School of Medicine, Department of Pathology, Molecular Pathology Laboratory, BIOREN-CEGIN, Temuco, Chile; 2 Pontificia Universidad Católica de Chile, School of Medicine, Department of Pathology, Santiago, Chile; 3 Department of Pathology, Hospital Hernán Henríquez Aravena, Temuco, Chile; 4 Department of Pathology, School of Medicine, Universidad de La Frontera, Manuel Montt 112 of 211, Temuco, Chile

**Keywords:** Epigenetics, Gallbladder cancer, Methylation, Tumor suppressor gene

## Abstract

Gallbladder cancer is an infrequent neoplasia with noticeable geographical variations in its incidence around the world. In Chile, it is the main cause of death owing to cancer in women over 40 years old, with mortality rates up to 16.5 per 100,000 cases. The prognosis is poor with few therapeutic options; in advanced cases there is only a 10% survival at 5 years.

Several studies mention the possible role of DNA methylation in gallbladder carcinogenesis. This epigenetic modification affects tumor suppressor genes involved in regulation pathways, cell cycle control, cell adhesion and extracellular matrix degradation, in a sequential and cumulative way. Determining DNA methylation patterns would allow them to be used as biomarkers for the early detection, diagnosis, prognosis and/or therapeutic selection in gallbladder cancer.

## Review

### Gallbladder cancer

Gallbladder cancer (GBC) is the most frequent malignant tumor of the biliary tract and the fifth most common cancer of the digestive tract. The presenting symptoms are vague, so diagnosis commonly occurs at an advanced stage. This late diagnosis combined with the fact that the gallbladder lacks a serosa result in a rather dismal prognosis [1-3]. The highest GBC incidence rates have been reported in women from India (21.5 out of 100,000), Chile (18.1 out of 100,000), Pakistan (13.8 out of 100,000) and Ecuador (12.9 out of 100,000). High incidences have also been found in Korea and Japan and some central and eastern European countries such as Poland, the Czech Republic and Slovakia [4]. These facts suggest significant genetic-environmental influences in the development of the disease [5].

Several factors have been associated with the risk of developing GBC. Lithiasis is one of the main risk factors, presenting in 65% to 90% of cases of GBC [2,3,6,7]; the risk is also associated with the number and size of the stones [8]. Likewise, and closely connected with lithiasis, chronic gallbladder inflammation might induce the continuous release of inflammatory mediators and growth factors (tumor promoters), which exert their effect on an epithelium previously damaged by carcinogenic agents [9]. Gallbladder cancer has also been associated with multiple familial polyposis (Gardner syndrome), Peutz-Jeghers syndrome, ‘porcelain’ gallbladder and anomalous pancreatobiliary ductal union [7].

**Figure 1:**
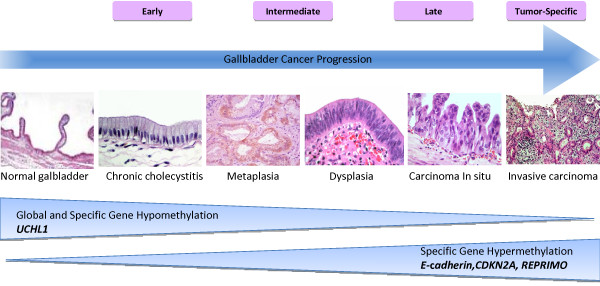
**Gallbladder cancer progression and its relationship to* E- *c* adherin *,* CDKN2A ( *cyclin-dependent kinase inhibitor 2A p16),* REPRIMO (TP53 dependent G2 arrest mediator candidate) *and* UCHL1 (ubiquitin carboxyl-terminal esterase L1) *methylation status*.*** The metaplasia frequently appears as an adaptive process to chronic inflammation generally produced by a gallstone. Dysplasia appears on top of this metaplasia, which progresses to a carcinoma *in situ* and subsequently becomes invasive. The progression of gallbladder cancer is accompanied by aberrant methylation of multiple genes and global hypomethylation. The first horizontal pyramid represents the decreasing methylation level in specific genes with oncogenic properties (*UCHL1*) that exhibits a methylated promoter region in normal cells, which can become activated in cancer cells by the loss of its methylation. Therefore, this pyramid shows the loss of global methylation and of this specific gene as the lesion progresses. The second horizontal pyramid represent methylation gain as the lesion progress; many tumor suppressor genes are silenced by DNA methylation during carcinogenesis (*E-cadherinCDKN2AREPRIMO*). Modified from "Preneoplastic lesions of a gallbladder from morphological and molecular points of view" 2007 [15]. With permission from Nova Science Publishers, Inc.

Adenocarcinoma is the most frequent histological type found in GBC. It represents 80% to 95% of all tumors, and the most frequent forms are moderately or poorly differentiated [1,2]. Two carcinogenic models of GBC sequence are recognized: the metaplasia-dysplasia-carcinoma and the adenoma-carcinoma, which have origins in two different types of epithelial lesion in the gallbladder. The metaplasia-dysplasia-carcinoma sequence, the most significant and frequent type of gallbladder carcinogenesis, is based on alterations to the epithelium of the gallbladder mucosa. The metaplasia frequently appears as an adaptive process secondary to chronic irritation or inflammation. Dysplasia appears on top of this metaplasia, which progresses to carcinoma *in situ* and subsequently becomes invasive (Figure 1). Severe dysplasia and carcinoma *in situ* have been found in more than 90% of GBC [10-12]. Less frequent is the second pathway (adenoma-carcinoma sequence), which suggests a malignant transformation from an adenomatous lesion [10,13]. The clinical and experimental evidence supports both models, and genetic-molecular studies show that the two pathways are distinct biological events [10,12,14].

Gallbladder carcinogenesis has been demonstrated to be a multifactorial process that involves genetic and ambient factors. For innovating early diagnosis, biomarkers and new therapeutic strategies, it is necessary to explore the molecular mechanisms of GBC development and progression, among them, epigenetic modifications.

### Epigenetics

Epigenetics is currently understood and defined as the inheritance of gene expression patterns not determined by the nucleotide sequence [16]. Alterations in the established epigenetic patterns generally lead to changes in gene expression, which can cause transcriptional repression [17]. The classic epigenetic modifications include DNA methylation, histone modification (acetylation, methylation, phosphorylation, and so on) and chromatin remodeling. A few years ago, a new epigenetic phenomenon was discovered, RNA interference, that has been shown to be involved in post-transcriptional silencing [18].

The most frequently studied epigenetic mechanism is DNA methylation, involved in the control of various biological processes in prokaryotes and eukaryote cells. In mammalian cells, DNA methylation only takes place at position 5' of the cytosine ring in cytosine-guanine dinucleotides (CpG) through the covalent bond of a methyl group [17,19]. Non-CpG sequences can also by methylated but with less frequency [20]. Generally, the CpG dinucleotide is poorly represented in the human genome, being approximately 1% of all nucleotide bases. The majority of these dinucleotides are dispersed throughout the genome in retrotransposon sequences and coding and non-coding gene regions. A lower percentage of CpG, little more than 15% of the total, is located in regions called CpG islets [21]. In approximately 60% of human genes, the CpG islets reside in promoters, first exons and 5′ untranslated region (5′UTRs) [22]. In normal cells, most dinucleotides located in repetitive regions of the genome (satellite DNA and endoparasitic elements) outside of CpG islets are methylated [23]. By contrast, the CpG islets are generally not methylated, except those located in the inactive chromosome X, in imprinted genes, specific germ-line genes, and in tissue-specific genes [24]. In normal tissue, methylation of some CpG islets usually increases with age, although the total genomic content of the methylcytosines decreases [25]. These same events are present in tumor cells, but are much more pronounced. The loss of methylation occurs in normally methylated CpG sites (in repetitive and/or endoparasitic sequences) and is known as overall genomic hypomethylation. The second alteration, characterized by an increase of methylation in CpG islets in normally demethylated regulatory regions, is known as aberrant hypermethylation. Many tumor suppressor genes are silenced by DNA methylation during carcinogenesis [26].

Genes that show oncogenic properties, that is, that exhibit a methylated promoter region in normal cells, can become reactivated in cancer cells by the loss of this methylation, resulting in hypomethylation [27]. In general, the chromosomal instability, loss of imprint, reactivation of transposable elements and transcriptional silencing of tumor suppressor genes or oncogene activation are of great benefit to the tumor cell [26].

### Methylation studies on the gallbladder

#### Aberrant methylation in preneoplastic gallbladder lesions

Chronic cholecystitis is an inflammatory disease associated with the presence of gallstones, which predetermines the appearance of the first morphological changes described in the metaplasia-dysplasia-carcinoma cancer sequence. House *et al.*[28] evaluated the hypermethylated state of six tumor-associated genes in a normal gallbladder, chronic cholecystitis and adenocarcinomas (samples fixed in formalin and embedded in paraffin) in a series of Chilean patients. In patients with chronic cholecystitis, they reported 28% methylation in some of the genes (*APCp16* and *hMLH1*), whereas only one case (5%) presented multigene methylation [28]. Likewise, García *et al.*[29] determined the gene methylation pattern in preneoplastic and neoplastic gallbladder lesions, finding that *DAPK-1DLC-1TIMP-3* and *RARβ-2* presented a progressive increase in their state of methylation from chronic cholecystitis to advanced carcinomas [29], reporting at the same time an aberrant methylation pattern of the gene for E-cadherin (*CDH1*) with a progressive increase in the methylation from chronic cholecystitis without metaplasia to advanced carcinoma (53% to 65.2%) [29]. Recently Shin *et al.*[30] reported that genes *TWIST1HOXA1SFRP1PENKGRIN2BCDH13NEUROG1TMEFF2TIMP3MINT2CCND2RASSF1ARUNX3DLC1CRABP1GATA3MT1GSEZ6LSOCS3THBS1* and *BCL2* were significantly methylated in extrahepatic cholangiocarcinoma (EHC) tissue samples, at higher levels than in cholecystitis and cholangitis tissues. This analysis was able to define a five-gene panel (*CCND2CDH13GRIN2BRUNX3* and *TWIST1*) in bile fluid samples capable of detecting EHC at a sensitivity of 83%, which was far higher than that of bile cytology (46%, *P* < 0.05) [30].

#### Aberrant methylation in gallbladder cancer

The methylation information available in cancers of the biliary tract is limited compared with other neoplasms. Nevertheless, studies have established that aberrant hypermethylation is an important event in the carcinogenesis of GBC (Table 1) [31-38]. Published studies have made it possible to establish that transcriptional gene silencing is due to the methylation state of its promoter regions, a mechanism that is alternative to mutation and allelic deletions. This seems to be an early, progressive and cumulative event in GBC, which increases from chronic cholecystitis without metaplasia to metaplasia. The variation of methylation frequencies in cases of different geographical origin, which suggests population differences, is worthy of note, because similar results have been observed in a study of genetic alterations (mainly mutations) [28]. For example, Takahashi *et al.*[32] reported in 2004 that, in Chilean patients, *SHP1* (80%), *3-OST-2* (72%), *CDH13* (44%), *P15INK4B* (44%), *CDH1* (38%), *RUNX3* (32%), *APC* (30%), *RIZ1* (26%), *P16INK4A* (24%) and *HPP1* (20%) presented a high percentage of methylation in patients with GBC (Table 1) [32]. For their part, García *et al.*[29] assessed the methylation state in *CDH13 (*69.6%), *DAPK1* (60.9%), *FHIT* (56.5%) and *RAR beta 2* (43.5%), genes which presented a high methylation frequency in advanced GBC in Chilean patients (Table 1). In addition, both of them found that the methylation state of *DLC1* was an indicator of poor prognosis, and methylation of *MGMT* is correlated with better survival [29]. Other authors evaluated the methylation states of *APC* and *FHIT* and their relationship to survival, with methylation percentages of 40% and 30%, respectively. No correlation was found between survival and methylation state [39]. Epigenetic inactivation by methylation in chromosome 3p is a frequent event in patients with GBC, particularly affecting the promoter region of the tumor suppressor genes *SEMA3B* (3p21.3) and *FHIT* (3p14.2) with 92% and 66% methylation, respectively [40]. *RASSF1A* inhibits the expression of the *RAS* oncogene, acting as a tumor suppressor gene through different pathways, including apoptosis, genomic stability and cell cycle regulation [41]. Epigenetic silencing in this gene has been reported in different human tumors, such as in lung, breast, brain, prostate, pancreas and kidney cancers [41]. In the gallbladder, it was found that the methylation in exon 1 of this gene was 36.4% in carcinoma samples, 25.0% in adenoma and 8.0% in normal epithelium [42,43]. When *RASSF1A* methylation was correlated with immunohistochemical expression, weak or no staining of the tumor cells was observed. The methylation frequency of this gene in cholangiocarcinoma can reach 65%, which, despite the close anatomical relationship with GBC, has different methylation patterns [44,45]. We also observed this situation in *RUNX3*, with 78.3% methylation in carcinoma of the biliary duct compared with 22.2% in GBC [43].

**Table 1:** Summary of the methylation rate of multiple genes studied in advanced gallbladder cancer

**Gene name**	**Full name**	**Function**	**Frequency of methylation % (n)**	**Origin of specimen**	**Method**	**Reference**
***CDH1***	*Cadherin 1, type 1*, *E-cadherin* (epithelial)	Tissue invasion (cell-cell adhesion)	11 (1/9)	Japan	MSP	Tozawa *et al.* 2004 [43]
			38 (19/50)	Chile	MSP	Takahashi *et al.* 2004 [32]
			65 (13/20)	Chile	MSP	Roa *et al.* 2006 [39]
			60 (13/20)	Chile	MSP	Roa *et al.* 2008 [46]
			65 (15/23)	Chile	MSP	García *et al.* 2009 [29]
			41 (9/22)	Japan	Nested MSP	Koga *et al.* 2005 [47]
***FHIT***	*Fragile histidine triad gene*	Regulation of DNA replication and apoptosis	30 (6/20)	Chile	MSP	Roa *et al.* 2006 [39]
			66 (33/50)	Chile	MSP	Riquelme *et al.* 2007 [40]
			32 (8/25)	Chile	MSP	Roa *et al.* 2008 [46]
			57 (13/23)	Chile	MSP	García *et al.* 2009 [29]
***APC***	*Adenomatous polyposis coli*	Cell migration, adhesion and apoptosis	26 (14/54)	Chile, USA	Nested MSP	House *et al.* 2003 [28]
			30 (15/50)	Chile	MSP	Takahashi *et al.* 2004 [32]
			40 (8/20)	Chile	MSP	Roa *et al.* 2006 [39]
			32 (8/25)	Chile	MSP	Roa *et al.* 2008 [46]
			35 (8/23)	Chile	MSP	García *et al.* 2009 [29]
***hMLH1***	*Human homologs of the MutL gene of bacteria*	Mismatch repair	13 (7/54)	Chile, USA	Nested MSP	House *et al.* 2003 [28]
			0 (0/9)	Japan	MSP	Tozawa *et al.* 2004 [43]
			5 (1/20)	Chile	MSP	Roa *et al.* 2006 [39]
			4 (2/50)	Chile	MSP	Riquelme *et al.* 2007 [40]
			4 (1/25)	Chile	MSP	Roa *et al.* 2008 [46]
			17 (4/23)	Chile	MSP	García *et al.* 2009 [29]
***p16***	*Cyclin-dependent kinase inhibitor 2A*	Cell cycle regulation	56 (30/54)	Chile, USA	Nested MSP	House *et al.* 2003 [28]
			60 (3/5)	Germany	MSP	Klump *et al.* 2003 [48]
			22 (2/9)	Japan	MSP	Tozawa *et al.* 2004 [43]
			24 (9/38)	Chile	MSP	Roa *et al.* 2004 [49]
			24 (12/50)	Chile	MSP	Takahashi *et al.* 2004 [32]
			15 (8/54)	China	MSP	Ueki *et al.* 2004 [31]
			20 (4/20)	Chile	MSP	Roa *et al.* 2006 [39]
			73 (37/51)	Japan	MSP	Tadokoro *et al.* 2007 [50]
			20 (5/25)	Chile	MSP	Roa *et al.* 2008 [46]
			26 (6/23)	Chile	MSP	García *et al.* 2009 [29]
***p15***	*Cyclin-dependent kinase inhibitor 2B*	Cell cycle regulation	44 (22/50)	Chile	MSP	Takahashi *et al.* 2004 [32]
			22 (5/23)	Chile	MSP	García *et al.* 2009 [29]
***DAPK1***	*Death-associated protein kinase 1*	Serine-threonine kinase	22 (2/9)	Japan	MSP	Tozawa *et al.* 2004 [43]
			8 (4/50)	Chile	MSP	Takahashi *et al.* 2004 [32]
			61 (14/23)	Chile	MSP	García *et al.* 2009 [29]
***DLC1***	*Deleted in liver cancer 1*	GTPase-activating protein	39 (9/23)	Chile	MSP	García *et al.* 2009 [29]
***RASSF1***	*RAS association domain family protein 1A*	Signal transduction	11 (1/9)	Japan	MSP	Tozawa *et al.* 2004 [43]
			0 (0/50)	Chile	MSP	Takahashi *et al.* 2004 [32]
			8 (4/50)	Chile	MSP	Riquelme *et al.* 2007 [40]
			36 (8/22)	Korea	MSP	Kee *et al.* 2007 [42]
			17 (4/23)	Chile	MSP	García *et al.* 2009 [29]
***MGMT***	*O-6-methylguanine-DNA methyltransferase*	Methyltransferase	13 (7/54)	Chile, USA	Nested MSP	House *et al.* 2003 [28]
			30 (7/23)	Chile	MSP	Garcia *et al.* 2009 [29]
***CDH13***	*Cadherin 13, H-cadherin* (heart)	Tissue invasion (cell-cell adhesion)	44 (22/50)	Chile	MSP	Takahashi *et al.* 2004 [32]
			70 (16/23)	Chile	MSP	García *et al.* 2009 [29]
***TIMP3***	*Metallopeptidase inhibitor 3*	Degradation of the extracellular matrix	0 (0/50)	Chile	MSP	Takahashi *et al.* 2004 [32]
			39 (9/23)	Chile	MSP	Garcia *et al.* 2009 [29]
***GSTP1***	*Glutathione S-transferase pi 1*	Conjugation of hydrophobic and electrophilic compounds	13 (3/23)	Chile	MSP	Garcia *et al.* 2009 [29]
***RARβ2***	*Retinoic acid receptor, beta*	Encodes retinoic acid receptor beta (mediates cellular signaling)	4 (2/54)	Chile, USA	Nested MSP	House *et al.* 2003 [28]
			14 (7/50)	Chile	MSP	Takahashi *et al.* 2004 [32]
			44 (10/23)	Chile	MSP	Garcia *et al.* 2009 [29]
***REPRIMO***	*TP53 dependent G2 arrest mediator candidate*	Cell cycle regulation (p53 mediator)	62 (31/50)	Chile	MSP	Takahashi *et al.* 2005 [51]
***SHP1***	*Protein tyrosine phosphatase, non-receptor type 6*	Regulate cell growth, differentiation, mitotic cycle	80 (40/50)	Chile	MSP	Takahashi *et al.* 2004 [32]
***3-OST-2***	*Heparan sulfate (glucosamine) 3-O-sulfotransferase 2*	Osulfotransferase	72 (36/50)	Chile	MSP	Takahashi *et al.* 2004 [32]
***RUNX3***	*Runt-related transcription factor 3*	TGF-beta signal pathway	22 (2/9)	Japan	MSP	Tozawa *et al.* 2004 [43]
			32 (16/50)	Chile	MSP	Takahashi *et al.* 2004 [32]
***RIZ1***	*PR domain containing 2, with ZNF domain*	Histone/protein methyltransferase	26 (13/50)	Chile	MSP	Takahashi *et al.* 2004 [32]
***HPP1***	*Transmembrane protein with EGF-like and two follistatin-like domains 2*	TGF-beta signal pathway	20 (10/50)	Chile	MSP	Takahashi *et al.* 2004 [32]
***P73***	*Tumor protein p73*	Induction of apoptosis and cell cycle regulation	28 (15/54)	Chile, USA	Nested MSP	House *et al.* 2003 [28]
			14 (7/50)	Chile	MSP	Takahashi *et al.* 2004 [32]
***SOCS-1***	*Suppressor of cytokine signaling 1*	JAK-STAT pathway	12 (6/50)	Chile	MSP	Takahashi *et al.* 2004 [32]
***DCR2***	*Tumor necrosis factor receptor superfamily, member 10d*	TNF-receptor superfamily	6 (3/50)	Chile	MSP	Takahashi *et al.* 2004 [32]
***SEMA3B***	*Sema domain, immunoglobulin domain (Ig), short basic domain, secreted, (semaphorin) 3B*	Induction of apoptosis	92 (46/50)	Chile	MSP	Riquelme *et al.* 2007 [40]
***DUTT1***	*Human homolog of Drosophila Roundabout (ROBO1)*	Cell migration and metastasis	22 (11/50)	Chile	MSP	Riquelme *et al.* 2007 [40]
***BLU***	*Zinc finger, MYND-type containing 10*	Cell cycle regulation	26 (13/50)	Chile	MSP	Riquelme *et al.* 2007 [40]
***UCHL1***	*Ubiquitin carboxyl - terminal esterase L1*	Peptidase C12 family	27 (6/22)	Korea	MSP	Lee *et al.* 2006 [52]
***p14***	*Ribonuclease P/MRP 14 kDa subunit*	Cell cycle regulation	40 (2/5)	Germany	MSP	Klump *et al.* 2003 [48]

JAK-STAT: janus kinase-signal transducer and activator of transcription; MSP: methylation specific PCR; TGF: transforming growth factor; TNF: tumor necrosis factor.

Recently it has been reported that *PSCA* is downregulated in non-neoplastic gallbladder lesions such as cholesterolosis, cholecystolithiasis and cholecystitis (9 out of 17; 53%). However, it was disclosed that the expression was decreased in more than 90% of cancers (40 out of 44) [53]. A DNA methylation assay revealed that the methylation level of the gene enhancer region was comparatively low in the cell lines of GBC with a relatively higher PSCA expression, suggesting that the methylation level of the region is related to the level of PSCA expression [53]. However, in the four GBC samples, no correlation was detected between the PSCA expression level and the DNA methylation level of the *PSCA* enhancer [53]. In others tumors, such as prostate and pancreatic cancers and gliomas, *PSCA* was recently reported to be upregulated [54], which suggests that PSCA has a different function dependent on the type of cancer. However, its function in normal and malignant epithelial cells is unknown.

### DNA promoter methylation of specific genes

Alterations in DNA methylation patterns are commonly found in all cancers, often with concomitant changes in gene expression. In GBC, molecular information is reduced; however, a high rate of methylation of some genes in GBC has been reported and associated with carcinogenesis of other tissues of the human digestive tract.

#### CDH1

The E-cadherin gene (*CDH1*), located on chromosome 16q22.1, is one of the most important tumor suppressor genes [55,56]. *CDH1* encodes for a transmembrane glycoprotein of 120 kDa that intervenes in cell adhesion mediated by calcium. It is a component of the E-cadherin/beta-catenin complex, which is important for cellular polarity, normal tissue morphology and cellular differentiation [56]. It also integrates adherens junctions (originally known as belt desmosomes) that form a continuous belt around the cell to bind the epithelial cells to each other and to maintain the integrity of the stratified epithelium [56]. *CDH1* belongs to a family of genes directly related to the processes of tumor invasion and cytoskeleton destabilization. CDH1 expression, it has been reported in less differentiated tumors, that generally have an unfavorable prognosis [57,58]. The most aggressive carcinomas generally show losses of epithelial cell cohesion, and this is often associated with a reduction in E-cadherin expression [59]. Some mutations of this gene have been detected in gastric, breast and endometrial cancer [60,61]. The methylation rate of different tumor suppressor genes in cancers of the digestive tract (stomach, colon, pancreas and gallbladder) has been studied, and show a high methylation frequency of *CDH1* (77.8%) [46]. *CDH1* has been described to be hypermethylated in GBC (Table 1), with frequencies that range from 11.1% to 65.2% [29,32,39,43,46,47]. Likewise, the methylation of this gene in advanced stage III and IV of GBC was evaluated, demonstrating approximately 60% methylation [46]. Previously, we reported that reduced expression of E-cadherin in resected gallbladder cancer tissues was significantly correlated with poor prognosis [62]. Loss of E-cadherin expression, followed by expression of the mts1 gene, may be an important event for increasing cell proliferation, motility and invasion activity in the progression of gallbladder cancer [63].

In relation to the degree of tumor differentiation, it was observed that *CDH1* presented a high methylation frequency in poorly differentiated tumors and in cases with three or more positive nodes [46]. A significant decrease in *CDH1* expression has been recorded as the lesion progresses, which may be due to methylation [39]. As far as patient survival is concerned, this gene does not exhibit significant differences in survival between the methylated and non-methylated cases [29,39].

Therefore, there is a significant correlation between the methylation of *CDH1* and the metastatic phenotype in GBC [32], which has already been described in previous studies on breast cancer [64].

Hypermethylation of *CDH1* has been reported in two cell lines of biliary tract cancer (SNU-478 and SNU-1079), with silenced mRNA expression. After treatment with a demethylating agent (5-aza-2′-deoxycytidine), *CDH1* was successfully re-expressed [65].

#### CDKN2A-p16

*CDKN2A-p16* is a tumor suppressor gene that encodes the protein p16, which is an inhibitor of cyclin-dependent kinase, involved in cell cycle regulation at Checkpoint G1. The loss of p16 expression is usually connected to homozygote deletion, loss of heterozygosity, mutations and methylation. A lower expression of p16 has been reported to increase the activity of D-type cyclin-dependent kinase activity, which translates into aberrant phosphorylation of the retinoblastoma gene, producing uncontrolled cell proliferation [31,46]. Loss of heterozygosity and homozygote deletion are two different pathways of p16 inactivation and have been shown to be combined with hypermethylation of the promoter in GBC [50]. In hepatocellular carcinoma and intrahepatic cholangiocarcinoma, *p16* is frequently inactivated by methylation of the promoter and rarely by deletion or mutation [31]. Inactivation of p16 through methylation of the promoter region has been frequently identified in breast, prostate, head and neck, liver, lung, brain, colon and esophageal cancers and cell lines of bladder cancer [66-69]*.* This is a tendency that is also observed in GBC with a loss of expression of up to 62.5% [48,50]. Takahashi *et al.*[32] reported that a considerable number of genes (8 out of 24 genes) are frequently methylated in GBC in comparison with chronic cholecystitis, varying in methylation frequency between 4% and 80%, with p16 having the lowest rate [32].

In 2006, Roa *et al.* published the methylation status of five genes in advanced carcinoma of the gallbladder and their correlation with the immunohistochemical expression, demonstrating that CDH1, APC, FHIT and CDKN2A might be important in the carcinogenesis of gallbladder [39]. Hypermethylation of *CDKN2A* is also one of the main mechanisms that induce the loss of *p16* expression [49]. Furthermore, Ueki *et al.* studied different alterations of *p16* in 68 tumors in Chinese patients, finding that only 14.8% presented aberrant hypermethylation [31]. Other investigations have identified a methylation percentage of 72.5% at different stages of progression; nevertheless, a significant relationship to the loss of expression of this protein was not established [50]. When the methylation state of this gene was evaluated in samples from the US and Chile, methylation frequencies of 56% were recorded, with similar methylation patterns in both populations [28].

In the analysis of *p16* and *p14,* it was noted that they presented a high methylation rate (between 40% and 60%) for carcinoma of the biliary duct, GBC and for primary sclerosing cholangitis. In normal tissue and choledocolithiasis, either no or an extremely low methylation rate was observed in the two genes [48].

The absence of alterations in *p16* (methylation, mutation, loss of heterozygosity in chromosome 9p) in cases of GBC means a better overall survival rate, and it is thus considered a significant prognostic factor [31]. A similar relationship has been seen in patients with stage IA non-small cell lung cancer, where the hypermethylation of *p16* was related to poorer survival [70]. This was not the case for other cancers (for example, of the biliary duct and the ampulla of vater), where the alteration had no influence on the prognosis [31].

#### Reprimo

This is a candidate tumor suppressor gene regulating *p53*, which is commonly altered in numerous human cancers. Reprimo is a highly glycosylated protein located in the cytoplasm that induces cell cycle arrest at the G2 phase, inhibiting the activity of Cdc2 and cyclin B1 [71,72]. The transcriptional repression of *Reprimo* by methylation was initially confirmed together with other genes in pancreatic cancer [72]. In addition, it was found to be hypermethylated in 16 different types of tumors, with a high percentage in gastric cancer (79%), gallbladder cancer (62%), lymphoma (57%), colorectal cancer (56%), esophageal adenocarcinoma (40%), breast cancer (37%) and leukemia (31%) [51]. As a result, it has been described as a potential biomarker for the early detection of gastric cancer [73].

Hypermethylation of this gene is infrequent in normal tissue; however, methylation percentages of 32% and 27% have been described in the gastric epithelium and colorectal polyps, respectively [51]. A low methylation pattern in close to 4% of cases has been described in chronic cholecystitis samples [51].

#### UCHL1

*UCHL1* (also known as *PGP9.5)* is the only gene with a potential oncogenic role that has found to be hypomethylated in the promoter region in GBC [52]. Is located on chromosome 4p14 and was identified originally as a member of a gene family whose products hydrolyze small C-terminal adducts of ubiquitin (Ub) to generate the ubiquitin monomer [74]*.* The product of the gene is a peptide responsible for eliminating Ub from proteins that have it, and to thereby avoid its degradation by the proteasome. Proteins degraded by this mechanism actively participate in cell cycle control, for example, p53 and a variety of cyclins [75]. Ub, a protein consisting of 76 amino acid residues, is present in all eukaryotic cells tested. It plays a role in the degradation of abnormal and short-lived proteins by the ATP- and Ub-dependent proteolytic systems. UCHL1 dysfunction in neurons is known to be involved in familial Parkinson’s disease [76]. *In vivo* analysis of *UCHL1*-deficient mice suggested that it functions as a regulator of apoptosis in neurons [77] and also in germinal cells during spermatogenesis [78].

The accumulation of Ub has been documented in several types of primary cancers [75]. During the past few years, several works have reported the link between UCHL1 expression and tumor progression, and that it may be useful as a potential marker of several human cancers, such as non-small cell lung cancer [79,80], invasive colorectal cancer [81], pancreatic cancer [82], squamous cell esophageal carcinoma [83] and neuroblastoma [84]. In GBC, a progressive decrease in the methylation of this gene has been observed, with 84.6% in normal epithelium, 37.5% in adenoma and 27.2% in carcinoma. These results suggest that hypomethylation of the *PGP9.5* promoter is a reliable marker in GBC and that DNA hypomethylation might play a significant role in the re-expression of the gene in GBC [52]. In addition, hypomethylation of *UCHL1* had previously been found in its promoter region in colorectal cancer, and lymph node metastasis was significantly associated with a lower frequency of methylation [85], poorer survival and a high incidence of recurrence [86].

## Discussion

DNA methylation is the epigenetic alteration most studied in the cancer cell. The number of genes with aberrant methylation in the human cancer cell is not known, but it is estimated that around 1% (or 250 genes) of the human genome can be aberrantly methylated in a tumor cell [87]. The use of methylation in the search for new biomarkers in GBC is a promising alternative since this epigenetic modification is an early, progressive and cumulative event in GBC. The methylation frequency of promoter regions of some important tumor suppressor genes, such as *p16CDH1REPRIMODAPK-1* and *SEMA3B*, is high in GBC [29,40,48,50,51,88], and has also been well documented in other cancers.

The tumor suppressor gene *p16* is frequently inactivated in a wide variety of human cancers by at least three distinct mechanisms: point mutation, small deletions of both *p16* alleles, and methylation of CpG islands [89]. Other studies have found methylation of the *p16* promoter in two of eight (25%) cholangiocarcinomas, and four of seven (57%) tumors analyzed by immunohistochemistry, demonstrated an absence of p16 nuclear staining in primary sclerosing cholangitis-associated cholangiocarcinoma [89]. Also, a high frequency of methylation (36 out of 72; 50%) has been reported in cases of intrahepatic and extrahepatic cholangiocarcinoma [45]. The expression of E-cadherin frequently diminishes as the tumor progresses, and abnormalities of E-cadherin expression have been associated with decreased apoptosis in GBC [88] and genomic instability during the process of neoplastic transformation [90]. In cholangiocarcinoma, mutations of *CDH1* are rare events [91]. Downregulation of E-cadherin expression is more commonly mediated through DNA methylation, with frequencies between 40% and 48% [45,92,93], while in GBC the frequency is between 11% and 65% [29,43]. This variation could be directly related to ethnic differences. Tadokoro *et al.*[50] found a frequency of 73% of methylation in p16 in samples from Japan, and Takahashi *et al.*[32] a frequency of 24% in samples from Chile. The age of the patients could also introduce variations in results. Methylation of *RUNX3* was more frequent in elderly patients [43]; environmental factors such as tobacco smoking [94] and *Helicobacter pylori* infection [95] can accelerate DNA methylation. *E-cadherin* methylation is an early event in gastric carcinogenesis, and is initiated by *H. pylori* infection [95]. Another possible reason for the variation could be differences in the methodology of study (MSP). House *et al.*[28] and Koga *et al.*[47] utilized a two-step MSP method, using nested PCR to increase the sensitivity of detecting allelic hypermethylation of targeted sequences and to facilitate the examination of multiple gene loci, and so the sensitivity in their studies tended to be higher than other studies that used single-step MSP [28]. All researchers used MSP technique for detection of DNA methylation, except Ono *et al.*[53], who used bisulfite-pyrosequencing in GBC cell lines.

MSP in its classical format is non-quantitative and cannot distinguish between low and high levels of a methylated target sequence. By contrast, combining real-time PCR probes with MSP, as in MethyLight assay, can achieve a quantitative assessment of the level of DNA methylation of a targeted sequence [96]. The effects of bisulfite treatment on DNA are difficult to control and often result in significant DNA degradation of up to 85% to 95% of target sequences [96], which would require the use of more sensitive techniques in the detection of potential biomarkers in clinical practice.

UCHL1 is a controversial molecule from an oncologic point of view. Hypomethylation of its promoter has been identified in a subset of human cancers, including GBC, presumably due to its intrinsic oncogenic properties or as a result of transformation. However, *UCHL1* has been reported to be repressed by methylation in other cancers, such as primary head and neck squamous cell carcinoma [97] and colorectal, ovarian [98] and pancreatic cancers [72]. Particularly, overexpression of UCHL1 has been found in pancreatic cancers [82] and it is associated with a poor prognosis, so the functional consequences of UCHL1 have yet to be determined.

## Conclusions

Chile is considered a very high-risk area for GBC, and its mortality rate reached 16.5 out 100,000 women in 2007 [99]. It is important to mention the paucity of examinations that enable the diagnosis of this neoplasia in the early stages, with vesicular lithiasis being the most serious risk factor in the metaplasia-dysplasia-carcinoma sequence. In the event of symptoms of acute cholecystitis, the main imaging finding that justifies the indication of elective or emergency cholecystectomy is lithiasis. In many cases, the cancer diagnosis is made based on findings in the anatomopathological examination of the surgical specimen. This explains the current health care policies in force in Chile since 2006, where elective cholecystectomy tends to be performed within guaranteed timeframes in women between 35 and 49 years of age who suffer from lithiasis, in an attempt to reduce the incidence of GBC or to increase the number of cases diagnosed at an early stage. This suggests the need to look for new methodologies that can assess the risk of progression to cancer in patients with preneoplastic lesions and/or clinical-morphological risk factors already known for GBC (such as female, obesity, ethnic group, cholecystolithiasis, gallstone volume, anomalous pancreaticobiliary junction, among others) [3].

Therefore, the study of gene methylation has at least five potential clinical applications: reactivating genes inactivated by methylation using demethylating drugs; identifying tumor cells in biological samples, allowing an early diagnosis since the change in methylation frequently precedes the appearance of advanced tumors; determining the methylation of individual genes or methylation profiles for groups of specific genes; being used as response markers to chemo- or hormone therapy; and allowing the transition between neoplastic and normal tissue to be determined in the surgical section margins according to the gene methylation profile [96,100,101]. Each of these, either separately or together, takes on special relevance in GBC, because they represent a problem that has scarcely been resolved in the three high-priority areas of oncology research: prevention, early diagnosis, and treatment.

Finally, the reversible nature of the epigenetic changes that occur in cancer must be mentioned as these have made epigenetic therapy possible as a treatment option. This therapy is based on reversing the epigenetic modifications that occur in tumor cells and re-establishing a normal epigenome. The possibility of reversing DNA methylation and reactivating the affected genes is an attractive option for a new therapeutic target in the treatment of cancer or preneoplastic lesions. However, the main problem with demethylating drugs is their poor specificity and they therefore cannot be used in the treatment of previously selected genes [102]. Nevertheless, satisfactory results have been reported regarding the use of epigenetic therapy in patients with myelodysplastic syndrome, approved by the Food and Drug Administration in 2004. This therefore opens a new therapeutic approach in the field of oncology that must be evaluated with multicentric clinical trials.

## Competing interests

The authors declare that they have no competing of interests.

## Authors’ contributions

PL designed the article and participated in writing all sections of the manuscript. PB revised the manuscript and contributed to content relating to methylation studies on the gallbladder cancer. OT contributed to content relating to conclusions and provided guidance for the structure. JCR provided guidance for the overall structure and content of the manuscript. All authors read and approved the final manuscript.
